# Salt-Sensitive Signaling Networks in the Mediation of K^+^/Na^+^ Homeostasis Gene Expression in *Glycyrrhiza uralensis* Roots

**DOI:** 10.3389/fpls.2017.01403

**Published:** 2017-08-14

**Authors:** Tao Lang, Shurong Deng, Nan Zhao, Chen Deng, Yinan Zhang, Yanli Zhang, Huilong Zhang, Gang Sa, Jun Yao, Caiwu Wu, Yanhong Wu, Qun Deng, Shanzhi Lin, Jianxin Xia, Shaoliang Chen

**Affiliations:** ^1^College of Life and Environmental Sciences, Minzu University of China Beijing, China; ^2^Beijing Advanced Innovation Center for Tree Breeding by Molecular Design, College of Biological Sciences and Technology, Beijing Forestry University Beijing, China; ^3^State Key Laboratory of Tree Genetics and Breeding, The Research Institute of Forestry, Chinese Academy of Forestry Beijing, China; ^4^College of Resource and Environmental Sciences, Hebei Normal University for Nationalities Chengde, China

**Keywords:** liquorice, ion flux, eATP, H_2_O_2_, NO, NaCl, NMT, RT-qPCR

## Abstract

We investigated the effects of salt-sensitive signaling molecules on ionic fluxes and gene expression related to K^+^/Na^+^ homeostasis in a perennial herb, *Glycyrrhiza uralensis*, during short-term NaCl stress (100 mM, 24 h). Salt treatment caused more pronounced Na^+^ accumulation in root cells than in leaf cells. Na^+^ ions were mostly compartmentalized in vacuoles. Roots exposed to NaCl showed increased levels of extracellular ATP (eATP), cytosolic Ca^2+^, H_2_O_2_, and NO. Steady-state flux recordings revealed that these salt-sensitive signaling molecules enhanced NaCl-responsive Na^+^ efflux, due to the activated Na^+^/H^+^ antiport system in the plasma membrane (PM). Moreover, salt-elicited K^+^ efflux, which was mediated by depolarization-activated cation channels, was reduced with the addition of Ca^2+^, H_2_O_2_, NO, and eATP. The salt-adaptive effects of these molecules (Na^+^ extrusion and K^+^ maintenance) were reduced by pharmacological agents, including LaCl_3_ (a PM Ca^2+^ channel inhibitor), DMTU (a reactive oxygen species scavenger), cPTIO (an NO scavenger), or PPADS (an antagonist of animal PM purine P2 receptors). RT-qPCR data showed that the activation of the PM Na^+^/H^+^ antiport system in salinized roots most likely resulted from the upregulation of two genes, *GuSOS1* and *GuAHA*, which encoded the PM Na^+^/H^+^ antiporter, salt overly sensitive 1 (SOS1), and H^+^-ATPase, respectively. Clear interactions occurred between these salt-sensitive agonists to accelerate transcription of salt-responsive signaling pathway genes in *G. uralensis* roots. For example, Ca^2+^, H_2_O_2_, NO, and eATP promoted transcription of *GuSOS3* (salt overly sensitive 3) and/or *GuCIPK* (CBL-interacting protein kinase) to activate the predominant Ca^2+^-SOS signaling pathway in salinized liquorice roots. eATP, a novel player in the salt response of *G. uralensis*, increased the transcription of *GuSOS3, GuCIPK*, *GuRbohD* (respiratory burst oxidase homolog protein D), *GuNIR* (nitrate reductase), *GuMAPK3*, and *GuMAPK6* (the mitogen-activated protein kinases 3 and 6). Moreover, *GuMAPK3* and *GuMAPK6* expression levels were enhanced by H_2_O_2_ in NaCl-stressed *G. uralensis* roots. Our results indicated that eATP triggered downstream components and interacted with Ca^2+^, H_2_O_2_, and NO signaling to maintain K^+^/Na^+^ homeostasis. We propose that a multiple signaling network regulated K^+^/Na^+^ homeostasis in NaCl-stressed *G. uralensis* roots.

## Introduction

Excess salts in the soil disrupts ion homeostasis in herbaceous and woody species (Munns and Tester, 2008; Polle and Chen, 2015). Maintaining cellular and whole-plant K^+^/Na^+^ homeostasis is required for plant adaptation to salt stress (Shabala et al., 2005; Sun et al., 2009a,b, 2010a,b; Chen and Polle, 2010; Chen et al., 2014). The plasma membrane (PM)-located H^+^-ATPase and Na^+^/H^+^ antiporter play crucial roles in maintaining K^+^/Na^+^ homeostasis in higher plants. The PM Na^+^/H^+^ antiporter, salt overly sensitive 1 (SOS1), prevents excessive Na^+^ accumulation in the cytoplasm (Zhu, 2001, 2016). The PM H^+^-ATPase sustains an H^+^ gradient to drive Na^+^ and H^+^ transport across the PM (Blumwald et al., 2000). Moreover, H^+^-pumps preserve a less-depolarized membrane potential, thus restricting K^+^ efflux through depolarization-activated outward rectifying K^+^ channels (DA-KORCs) and non-selective cation channels (DA-NSCCs, Sun et al., 2009b, 2012a; Zhang et al., 2015). A large body of evidence suggests that salt-sensitive signaling molecules, such as extracellular ATP (eATP), hydrogen peroxide (H_2_O_2_), calcium (Ca^2+^), nitric oxide (NO), and their crosstalk contribute to the regulation of the Na^+^/H^+^ antiport system (the H^+^-ATPase and Na^+^/H^+^ antiporter). This system contributes to K^+^/Na^+^ homeostasis in a variety of plant species (Zhang et al., 2007; Chen et al., 2010; Sun et al., 2010a,b, 2012a; Lu et al., 2013; Lang et al., 2014).

Salt-elicited cytosolic Ca^2+^ upregulates PM Na^+^/H^+^ antiporter activity via the SOS-signaling pathway in Arabidopsis (Qiu et al., 2002; Zhu, 2003), rice (Martínez-Atienza et al., 2007), and poplar (Tang et al., 2010). H_2_O_2_ induces the entry of Ca^2+^ through PM Ca^2+^-permeable channels (Pei et al., 2000; Mori and Schroeder, 2004), and this mechanism was suggested to trigger the Ca^2+^-SOS pathway (Sun et al., 2010b). NO functions as a gaseous signaling molecule, which induces resistance to salt injury by depleting the Na^+^ content, as previously shown in reed callus (Zhang et al., 2006) and in salt-secreting and non-secreting mangroves (Chen et al., 2010; Lu et al., 2013; Lang et al., 2014). Extracellular ATP acts as a signaling molecule and plays a significant role in protecting against NaCl stress (Kim et al., 2009; Sun et al., 2012a; Chen et al., 2014; Lang et al., 2014; Polle and Chen, 2015). It is suggested that eATP can be sensed by a purinergic ATP (P2) receptor in the PM, most likely P2K1 (Choi et al., 2014), and P2 receptor binding induces downstream signaling components, e.g., H_2_O_2_ and Ca^2+^ (Demidchik et al., 2009; Sueldo et al., 2010; Sun et al., 2010b, 2012a). Indeed, eATP interacted with H_2_O_2_ and Ca^2+^ to induce resistance to Na^+^ toxicity in mangrove roots (Lang et al., 2014). However, the effect of eATP signaling cascades on Na^+^ homeostasis remains to be elucidated in salt-resistant herbaceous species, e.g., *Glycyrrhiza uralensis*.

NaCl exposure caused membrane depolarization and net K^+^ efflux in Arabidopsis (Shabala et al., 2005, 2006), barley (Shabala et al., 2003; Chen et al., 2007), *Populus euphratica* (Sun et al., 2009b, 2010a,b, 2012a; Zhao et al., 2016), and mangrove species (Chen et al., 2010; Lu et al., 2013; Lang et al., 2014). Ca^2+^ blocked NaCl-induced K^+^ loss, which was mediated by depolarization-activated KORCs and NSCCs in Arabidopsis (Shabala et al., 2006) and in poplars (Sun et al., 2009b). This was mainly due to the activated PM H^+^-ATPase, which lowers the NaCl-depolarized membrane potential, thus restricting K^+^ loss through KORCs and NSCCs (Shabala et al., 2006; Sun et al., 2009b). H_2_O_2_, NO, and eATP were also shown to maintain K^+^ homeostasis by up-regulating PM proton pumps in poplar species (Zhang et al., 2007; Sun et al., 2010a,b, 2012a; Zhao et al., 2016) and mangroves (Chen et al., 2010; Lu et al., 2013; Lang et al., 2014). However, interactions between these stress signaling molecules in the regulation of K^+^ homeostasis remains to be established in liquorice plants.

*Glycyrrhiza uralensis* Fisch. (Licorice), a perennial herb of the genus Leguminosae, is naturally distributed in the arid and semi-arid areas of eastern Asia (Li et al., 2016). Licorice is frequently used as a crude therapeutic medicine to protect against multiple diseases in Asian populations (Mochida et al., 2017). Apart from its pharmaceutical functions, *G. uralensis* is ecologically important, both for conserving soil and water and for improving soil structure in semiarid ecosystems (Zhang and Ye, 2009). The deep-rooted nature of *G. uralensis* plants enables them to survive desert and semi-desert habitats in northwestern China. However, how *G. uralensis* sustains ionic homeostasis under saline conditions and whether salt-sensitive signals contribute to the demonstrated salt tolerance have not been investigated in this liquorice species.

In the present study, we aimed to characterize the importance of Ca^2+^, H_2_O_2_, NO, and eATP in mediating Na^+^/H^+^ transport in the salinized roots of *G. uralensis*. Flux measurements with non-invasive micro-test technology (NMT) revealed that these salt-induced signals were essential for restricting K^+^ efflux and enhancing Na^+^ exclusion in liquorice roots. We also screened for alterations in the transcription of genes involved in various salt-signaling pathways. We aimed to explore the network of multiple interactions among Ca^2+^, H_2_O_2_, NO, and eATP in the regulation of signaling and gene expression related to K^+^/Na^+^ homeostasis in *G. uralensis* roots.

## Materials and Methods

### Plant Materials and Culture Conditions

Seeds of *G. uralensis* were obtained from the Mongolian Autonomous County of Hoboksar, Tarbagatay Prefecture, Xinjiang Uygur Autonomous Region (latitude 46°82′N, longitude 85°75′E). The seeds were planted in plastic pots (5 cm in diameter, 8 cm in height), containing a 2:1 mixture of sand and nursery soil, and placed in a growth chamber at Beijing Forestry University, Beijing, China. The potted *G. uralensis* were well irrigated, according to evaporation demand, and fertilized with one-quarter-strength Hoagland solution weekly. The temperature and relative humidity were maintained at 25–28°C and 60–70%, respectively. A photoperiod of 14 h (9:00–23:00) was applied, and photosynthetically active radiation varied from 280 to 350 μmol m^-2^s^-1^. After 2 weeks of culture, rooted liquorice seedlings were transferred to 300-ml pots containing one-quarter-strength Hoagland’s nutrient solution for hydroponic equilibration.

### Salt Treatments

Hydroponic-equilibrated seedlings of *G. uralensis* were subjected to 0 or 100 mM NaCl for 24 h. Na^+^ concentrations in root and leaf cells were examined after 6, 12, and 24 h of treatment. Na^+^, K^+^, and H^+^ fluxes were measured along the root axes with the NMT technique. The effects of PM transporter/channel inhibitors were examined in NaCl-treated *G. uralensis*. A blocker of the Na^+^/H^+^ antiporter, amiloride (50 μM), and a specific inhibitor of the H^+^-ATPase, sodium orthovanadate (500 μM), were used to inhibit the Na^+^/H^+^ antiport system in the PM (Sun et al., 2009a). A typical K^+^ channel inhibitor, tetraethylammonium chloride (TEA, 50 μM), was used to reduce NaCl-elicited K^+^ efflux (Lu et al., 2013; Lang et al., 2014). In our study, control and NaCl-treated roots were treated with these inhibitors for 30 min before the flux recordings. In addition, two series of experiments (described below) were carried out to determine the involvement of Ca^2+^, H_2_O_2_, NO, and eATP in regulating Na^+^ and K^+^ fluxes and gene expression in NaCl-treated *G. uralensis* roots.

### Series 1: Agonist Treatments

We added exogenous agonists, CaCl_2_ (10 mM), H_2_O_2_ (10 mM), the NO donor, sodium nitroprusside (SNP, 100 μM), and ATP-Na_2_ (300 μM), and measured the effects on NaCl-induced Na^+^ and K^+^ fluxes in young roots of *G. uralensis*. The chemicals were added to one-quarter-strength nutrient solution in the presence and absence of NaCl (100 mM). Control plants treated with or without salt were cultured in nutrient solution without the application of the chemicals mentioned above. The steady-state fluxes of K^+^ and Na^+^ were recorded along the root axis after 24-h NaCl treatments.

We also examined the expression levels of genes involved in salt transport and signaling after salt and agonist (Ca^2+^, H_2_O_2_, SNP, and ATP) treatments. Specifically, we examined expression of the PM H^+^-ATPase gene, *GuAHA*; the PM Na^+^/H^+^ antiporter gene, *GuSOS1*; the salt overly sensitive 3 gene, *GuSOS3*; the calcineurin B-like protein (CBL)-interacting protein kinase gene, *GuCIPK*; the respiratory burst oxidase homolog protein D gene, *GuRbohD*; the nitrate reductase gene, *GuNIR*; and the mitogen-activated protein kinases 3 and 6 genes, *GuMAPK3* and *GuMAPK6*.

### Series 2: Antagonist Treatments

Control and NaCl (100 mM, 24 h)-stressed *G. uralensis* seedlings were treated with or without pharmacological agents for 30 min. These agents were: LaCl_3_, an inhibitor of the PM Ca^2+^ channel (5 mM); DMTU, a ROS scavenger (5 mM); cPTIO, a scavenger of NO (300 μM); and PPADS, an antagonist of animal PM P2 receptors (300 μM) (Sun et al., 2010a,b; Chen et al., 2013; Zhao et al., 2016). Next, young roots with apices of 2.0–3.0 cm were sampled and equilibrated in measuring solution for 30 min. Then, steady-state fluxes of K^+^ and Na^+^ along the root axes were recorded in plants after treating with NaCl and antagonist (LaCl_3_, DMTU, cPTIO, and PPADS). We also examined the abundances of *GuAHA* and *GuSOS1* transcripts in these roots.

### Protocols for NMT Recording

We used the NMT technique (NMT-YG-100, Younger United States LLC, Amherst, MA, United States) to measure the net Na^+^, K^+^, and H^+^ fluxes in *G. uralensis* roots. The microelectrodes were prepared and calibrated as previously described (Sun et al., 2009a,b; Lang et al., 2014).

After roots were exposed to NaCl treatment, with either an agonist (Ca^2+^, H_2_O_2_, SNP, and ATP) or an antagonist (amiloride, sodium orthovanadate, TEA, LaCl_3_, DMTU, cPTIO, and PPADS), root segments with 2.0–3.0 cm apices were selected and washed two or three times with redistilled water. When placed in a buffer with a lower Na^+^ concentration, the preloaded Na^+^ would diffuse from the surface of salt-stressed roots. To decrease the effect of this excess salt release on flux recordings, roots were equilibrated prior to flux recordings in a measuring solution (0.1 mM NaCl, 0.1 mM MgCl_2_, 0.1 mM CaCl_2_, and 0.5 mM KCl) for 30 min. The concentrations of Ca^2+^ and K^+^ in the measuring solution were set to 0.1 and 0.5 mM, respectively (Li et al., 2012), to reduce interference from Ca^2+^ and K^+^ on the Na^+^ electrodes (Cuin et al., 2011). The pH of the measuring solution was adjusted to 5.7 with HCl and KOH.

After equilibration, roots were immobilized on the bottom of a measuring chamber with 10 ml of fresh measuring solution. Flux measurements were started at 200 μm from the root apex and conducted along the root axis, up to 2700 μm from the root apex, at intervals of 200 or 300 μm (vigorous ion fluxes were typically observed at the apical regions; Lu et al., 2013; Lang et al., 2014). A 6–8 min continuous recording was performed at each measuring point in the apical zones. Five or six individual seedlings were measured from each treatment group.

### Na^+^ Visualization within Root and Leaf Cells

To evaluate the NaCl-induced Na^+^ distribution in *G. uralensis* roots and leaves, we used a specific fluorescent probe, CoroNa-Green AM (Sun et al., 2012a). Two-week-old seedlings were exposed to 0 or 100 mM NaCl for 6, 12, or 24 h. Then, the roots and leaves were exposed to CoroNa-Green AM (20 μM) for 2 h in a 5 mM Mes-KCl loading buffer (pH 5.7). Cellular Na^+^ was visualized with a Leica SP5 confocal microscope (Leica Microsystems GmbH, Wetzlar, Germany). The confocal settings were as follows: excitation 488 nm, emission 510–530 nm, frame = 512 × 512.

### Cytosolic Ca^2+^, H_2_O_2_, and NO Levels in Roots

In *G. uralensis* roots, we used specific fluorescent probes to detect cellular signal contents. We used Rhod-2 AM (Biotium) to detect cytosolic Ca^2+^ (Sun et al., 2012a; Zhang et al., 2015); H_2_DCF-DA (Eugene) to detect H_2_O_2_ (Sun et al., 2010a,b); and DAF-FM DA (Eugene) to detect NO (Sun et al., 2012a). Briefly, young roots were exposed to 0 or 100 mM NaCl for 30 min. Then, the roots were transferred to a 5 mM Mes-KCl loading buffer (pH 5.7) containing 2 μM Rhod-2 AM, 50 μM H_2_DCF-DA, or 10 μM DAF-FM DA. The staining was performed in the dark for 1 h at room temperature. Next, the roots were washed 4–5 times with Murashige and Skoog (MS) liquid medium prior to confocal microscope measurements. The confocal settings were as follows: excitation 488 nm, emission 510–530 nm for H_2_DCF-DA and DAF-FM DA; and excitation 543 nm, emission 570–590 nm for Rhod-2 AM (frame = 512 × 512).

### Extracellular ATP in Roots

Extracellular ATP levels were monitored with the Enlighten ATP assay system bioluminescence kit (Promega, Madison, WI, United States; Sun et al., 2012a; Deng et al., 2015). Briefly, *G. uralensis* roots were exposed to 0 or 100 mM NaCl at room temperature. The liquid culture medium of control and NaCl-treated roots was sampled at 0, 5, 10, 20, 40, 60, 120, and 240 min, then immediately frozen in liquid nitrogen. eATP was measured in an assay with luciferin-luciferase Turner Designs Modulus^TM^ Microplate Multimode Reader (Promega Corp., Madison, WI, United States). The eATP levels were calculated, based on a standard curve created by measuring a linear range (0.01–100 nM) of standard eATP concentrations (Sun et al., 2012a; Deng et al., 2015).

### Quantitative Real-time PCR Analysis

The transcription levels of genes related to the PM Na^+^/H^+^ transport system and salt signaling were evaluated in salt-stressed plants. Quantitative real-time PCR assays were conducted according to Deng et al. (2015) with some modifications. Briefly, total RNA was isolated from *G. uralensis* roots with TRIzol reagent (Invitrogen). DNA was eliminated by treating for 0.5 h with DNase I (Promega). An aliquot of purified RNA (1 μg) was used as template for first strand cDNA synthesis with M-MLV reverse transcriptase (Promega) and oligo (dT) primers. Specific primers for *GuAHA*, *GuSOS1*, *GuSOS3*, *GuCIPK*, *GuRbohD*, *GuNIR*, *GuMAPK3*, and *GuMAPK6* were designed, based on homologous sequences found in *Populus trichocarpa* or Arabidopsis. Forward and reverse primers are listed in Supplementary Table 1. Amplification was performed as described by Ding et al. (2010): 95°C for 5 min, followed by 32 cycles of 94°C for 30 s, 55°C for 30 s, and finally, 72°C for 30 s, with a final step of 72°C for 10 min. The transcripts of target genes were normalized to the expression level of the *G. uralensis* β-actin 2 gene (*GuACT2*), and relative expression was calculated with the 2^-ΔΔC_T_^ method (Livak and Schmittgen, 2001). Each experiment was replicated at least three times, and mean values are shown.

### Data Analysis

Ion fluxes were evaluated with JCal V3.0, which was created by Yue Xu^1^. In the present study, positive values denote cation efflux and negative values denote cation influx. All experimental data were processed with SPSS 17.0 for statistical tests. Data were subjected to an Analysis of Variance (ANOVA), and comparisons between means were performed with Duncan’s multiple range test. *P*-values less than 0.05 were considered statistically significant.

## Results

### Na^+^ Levels in Root and Leaf Cells

**FIGURE 1:**
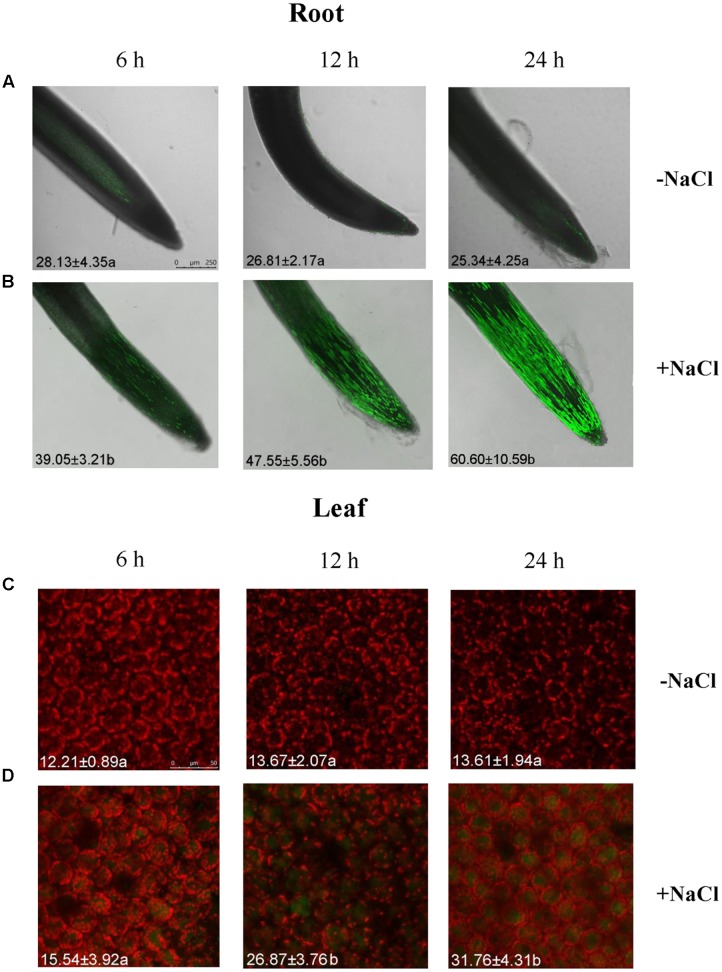
Na^+^ concentrations in root and leaf cells of NaCl-stressed *Glycyrrhiza uralensis*. Two-week-old *G. uralensis* seedlings were exposed to 0 or 100 mM NaCl for 6, 12, and 24 h in one-quarter-strength Hoagland solution, then stained with CoroNa-Green AM to detect Na^+^ concentrations. Representative confocal images of **(A,B)** roots (scale bar: 250 μm) and **(C,D)** leaves (scale bar: 50 μm) show the Na^+^ content (bright green fluorescence). The orange-red color is chlorophyll autofluorescence. The mean value (±SD) of 4–5 independent experiments is shown in the left bottom corner of each panel, and different letters (a and b) denote significant differences (*P* < 0.05) between control (–NaCl) and salt treatment (+NaCl).

The Na^+^ concentrations in roots and leaves of *G. uralensis* were detected with a Na^+^-sensitive fluorescent dye, CoroNa-Green AM. The Na^+^ fluorescence in roots and leaves increased with the duration of salt exposure (6, 12, and 24 h) (**Figure 1**). Intracellular Na^+^ was detected as a bright green fluorescence, which was typically observed in vacuoles (**Figure 1**). However, Na^+^ levels in roots were 1.69- to 2.40-fold higher than that in leaves over the observation period (**Figure 1**). This result indicated that *G. uralensis* roots could take up and accumulate high Na^+^ within a short period of salt treatment. Therefore, the roots were used to evaluate the effects of salt signaling molecules on ion fluxes and gene transcription.

### NaCl-Elicited Signaling Molecules in *G. uralensis* Roots

**FIGURE 2:**
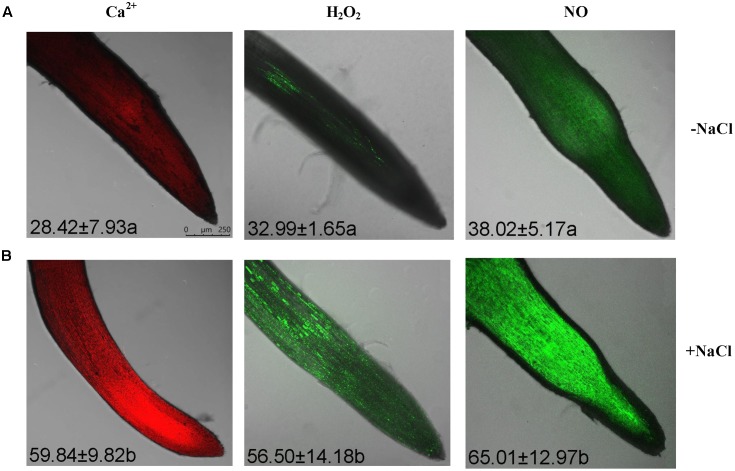
Cytosolic Ca^2+^, H_2_O_2_, and NO levels within root cells in NaCl-stressed *G. uralensis*. Two-week-old *G. uralensis* seedlings were exposed to 0 or 100 mM NaCl for 30 min in one-quarter-strength Hoagland solution, then stained with specific fluorescent probes for detecting Ca^2+^ (Rhod-2, orange-red), H_2_O_2_ (H_2_DCF, green), and NO (DAF-FM, green). Representative confocal images (scale bar: 250 μm) show **(A)** control and **(B)** NaCl-stressed roots. The bright green fluorescence corresponded to the detection of H_2_O_2_ and NO, while the orange-red color is the Ca^2+^ fluorescence. The mean value (±SD) of 4–5 independent experiments is shown in the left corner of each panel, and different letters (a and b) denote significant differences (*P* < 0.05) between control (–NaCl) and salt treatment (+NaCl).

**FIGURE 3:**
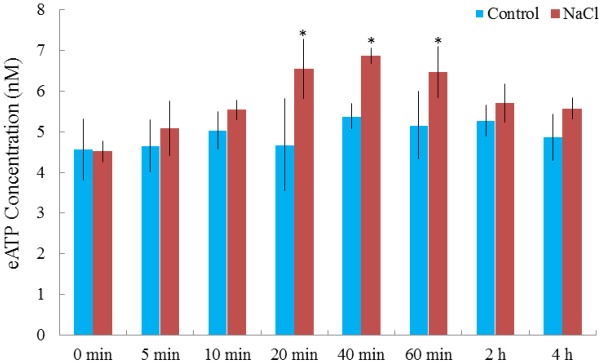
NaCl-induced alterations in extracellular ATP (eATP) in *G. uralensis* roots. Two-week-old *G. uralensis* seedlings were exposed to 0 or 100 mM NaCl for 4 h in one-quarter-strength Hoagland solution. Extracellular ATP was detected with the Enlighten ATP assay system bioluminescence kit. Each value (±SD) is the mean of 4–5 independent experiments. ^∗^*P* < 0.05, compared to no-salt control.

Rhod-2 AM, H_2_DCF-DA, and DAF-FM DA, respectively, were used to detect cytosolic Ca^2+^, H_2_O_2_, and NO elicited by NaCl in *G. uralensis* roots (Sun et al., 2010a,b, 2012a). Confocal assays (**Figure 2**) revealed that cytosolic Ca^2+^ (color: pseudo-red), H_2_O_2_ (color: pseudo-green), and NO (color: pseudo-green) significantly increased by 71–111% after a 30 min salt shock. Similarly, in an ATP-bioluminescence assay, NaCl caused a marked rise in eATP after 20 min of stress, and the peak level occurred at 40 min of stress (**Figure 3**).

### Effect of Signaling Molecules on NaCl-Induced Ion Fluxes

#### Na^+^ Flux

**FIGURE 4:**
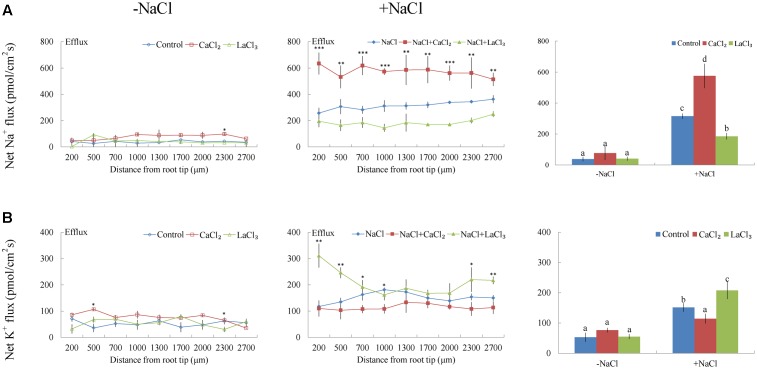
Effects of CaCl_2_ and LaCl_3_ on Na^+^ and K^+^ fluxes in *G. uralensis* roots under salt stress. Roots were untreated (control, blue) or exposed to CaCl_2_ (10 mM, red) for 24 h in the absence (–NaCl) and presence of NaCl (100 mM). For inhibitor treatment, control and NaCl-stressed roots were subjected to LaCl_3_ (5 mM, green) for 30 min. Steady-state flux profiles of **(A)** Na^+^ and **(B)** K^+^ were measured along the root axis at the apical zones (200–2700 μm from the root tip) in no-salt (*left panels*) and salt-stressed (*center panels*) conditions. Each point represents the mean of five to six individual plants. ^∗^*P* < 0.05, ^∗∗^*P* < 0.01, ^∗∗∗^*P* < 0.001, compared to controls. (*Right panels*) Means of Na^+^ and K^+^ fluxes at all measurement points, in no-salt (–NaCl) and salt-stressed (+NaCl) plants. Bars (±SD) represent the means of five to six individual plants; different letters (a, b, c, and d) indicate significant differences (*P* < 0.05) between treatments.

Under no-salt control conditions, *G. uralensis* roots exhibited stable, constant Na^+^ efflux along the root apex, with a mean value of 37.89 pmol cm^-2^ s^-1^ (**Figure 4A**). After exposure to NaCl (100 mM) for 24 h, Na^+^ efflux along the root tip significantly increased to 315.24 pmol cm^-2^ s^-1^ (**Figure 4A**). Of note, the maturation region (1700–2000 μm from the apex) displayed 10–20% higher Na^+^ efflux than the meristematic zone (200 μm from the apex).

Under NaCl exposure, the addition of 10 mM Ca^2+^ markedly increased the Na^+^ efflux by 82% in the measured root regions (**Figure 4A**). However, the addition of LaCl_3_ (5 mM), an inhibitor of Ca^2+^-channels in the PM, markedly reduced the salt-elicited Na^+^ efflux (**Figure 4A**). Compared to NaCl treatment, in no-salt control conditions, exogenously applied CaCl_2_ or LaCl_3_ had no significant effect on root Na^+^ flux with the exception of a few measuring points (**Figure 4A**).

**FIGURE 5:**
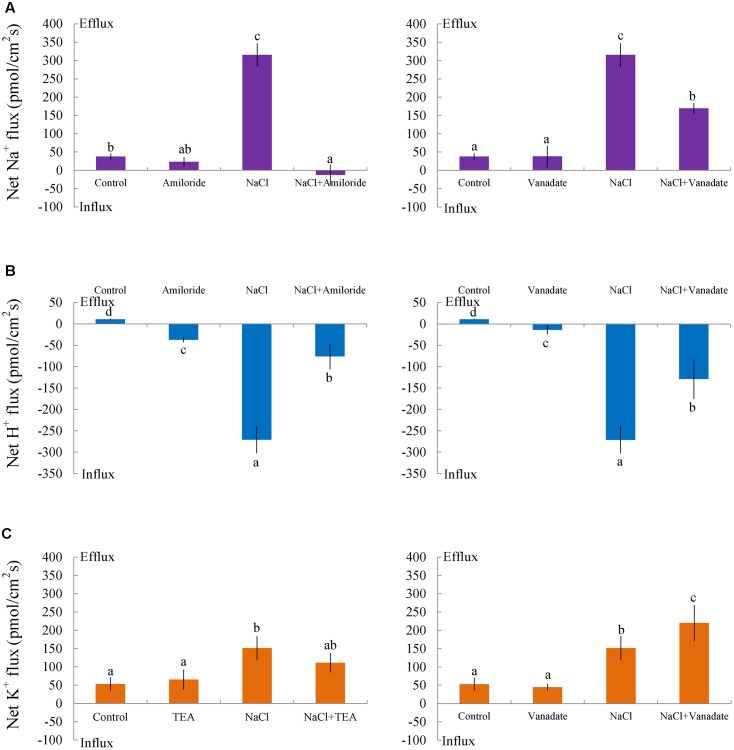
Effects of amiloride, sodium orthovanadate, and tetraethylammonium (TEA) on Na^+^, K^+^, and H^+^ fluxes in *G. uralensis* roots under salt stress. Roots were exposed to 0 (control) or 100 mM NaCl (NaCl) for 24 h, then exposed to transporter/channel inhibitors for 30 min. Steady-state fluxes were measured along the root axis at the apical zones (200–2700 μm from the root tip). Mean fluxes of **(A)** Na^+^ and **(B)** H^+^ were measured in the absence and presence of inhibitors, (*left*) amiloride (50 μM) and (*right*) sodium orthovanadate (500 μM). **(C)** The mean K^+^ flux was measured in the absence and presence of inhibitors, (*left*) TEA (50 μM) and (*right*) sodium orthovanadate (500 μM). Bars (±SD) represent the means of five to six individual plants; letters (a, b, c, and d) indicate significant differences between treatments (*P* < 0.05).

Pharmacological experiments revealed that salt-elicited Na^+^ efflux was significantly suppressed by amiloride (an inhibitor of the Na^+^/H^+^ antiporter) or sodium orthovanadate (a specific inhibitor of the PM H^+^-ATPase) (**Figure 5A**). Moreover, steady-state recordings showed that these inhibitors markedly decreased the H^+^ influx induced by salt treatment (**Figure 5B**). These results indicated that salt-stimulated Na^+^ efflux was due to active Na^+^ extrusion, i.e., Na^+^/H^+^ antiport across the PM, in this medicinal plant.

**FIGURE 6:**
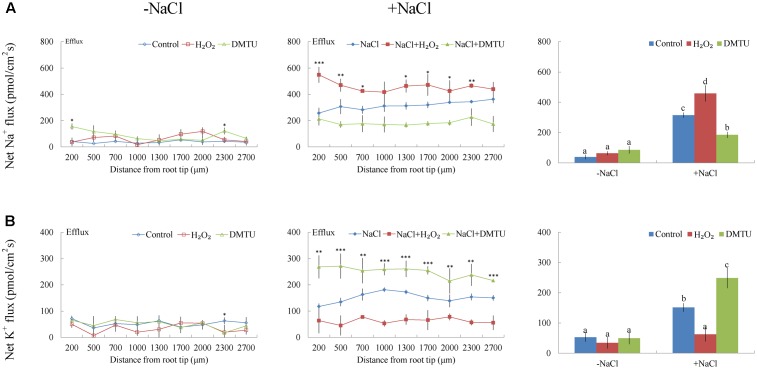
Effects of H_2_O_2_ and DMTU on Na^+^ and K^+^ fluxes in *G. uralensis* roots under salt stress. Roots were untreated (control, blue) or exposed to H_2_O_2_ (10 mM, red) for 24 h in the absence (–NaCl, no-salt) and presence of NaCl (100 mM). For inhibitor treatment, no-salt and NaCl-stressed roots were subjected to DMTU (5 mM, green) for 30 min. Steady-state flux profiles of **(A)** Na^+^ and **(B)** K^+^ were measured along the root axis at the apical zones (200–2700 μm from the root tip) in no-salt (*left panels*) and salt-stressed (*center panels*) conditions. Each point represents the mean of five to six individual plants. ^∗^*P* < 0.05, ^∗∗^*P* < 0.01, and ^∗∗∗^*P* < 0.001, compared to controls. (*Right panels*) Bars (±SD) represent the mean of five to six individual plants; letters (a, b, c, and d) indicate significant differences (*P* < 0.05) between treatments.

**FIGURE 7:**
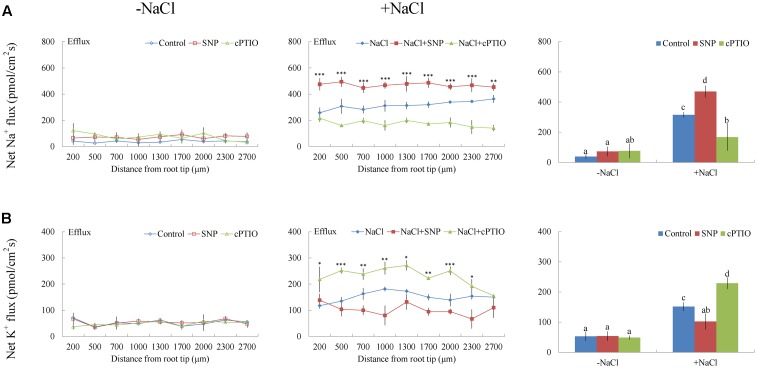
Effects of sodium nitroprusside (SNP) and cPTIO on Na^+^ and K^+^ fluxes in *G. uralensis* roots under salt stress. Roots were untreated (control, blue) or exposed to SNP (100 μM, red) for 24 h in the absence (–NaCl, no-salt) and presence of NaCl (100 mM). For inhibitor treatment, no-salt and NaCl-stressed roots were subjected to cPTIO (300 μM, green) for 30 min. Steady-state flux profiles of **(A)** Na^+^ and **(B)** K^+^ were measured along the root axis at the apical zones (200–2700 μm from the root tip) in no-salt (*left panels*) and salt-stressed (*center panels*) conditions. Each point represents the mean of five to six individual plants. ^∗^*P* < 0.05, ^∗∗^*P* < 0.01, ^∗∗∗^*P* < 0.001, compared to controls. (*Right panels*) Bars (±SD) represent the mean of five to six individual plants; letters (a, b, c, and d) indicate significant differences (*P* < 0.05) between treatments.

**FIGURE 8:**
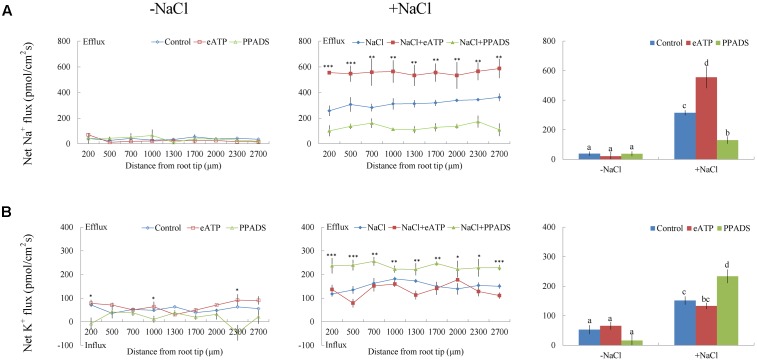
Effects of eATP and PPADS on Na^+^ and K^+^ fluxes in *G. uralensis* roots under salt stress. Roots were untreated (control, blue) or exposed to ATP-Na_2_ (300 μM, red) for 24 h in the absence (–NaCl, no-salt) and presence of NaCl (100 mM). For inhibitor treatment, no-salt and NaCl-stressed roots were subjected to PPADS (300 μM, green) for 30 min. Steady-state flux profiles of **(A)** Na^+^ and **(B)** K^+^ were measured along the root axis at the apical zones (200–2700 μm from the root tip) in no-salt (*left panels*) and salt-stressed (*center panels*) conditions. Each point represents the mean of five to six individual plants. ^∗^*P* < 0.05, ^∗∗^*P* < 0.01, and ^∗∗∗^*P* < 0.001, compared to controls. (*Right panels*) Bars (±SD) represent the mean of five to six individual plants; letters (a, b, c, and d) indicate significant differences (*P* < 0.05) between treatments.

Under short-term NaCl stress, exogenously applied H_2_O_2_ (10 mM), SNP (a NO donor, 100 μM), or ATP (300 μM) produced an effect similar to that of CaCl_2_ (**Figures 6A**–**8A**). More pronounced effects were observed with ATP treatment, which induced a mean Na^+^ flux of 555.86 pmol cm^-2^ s^-1^, compared to fluxes of 458.84 pmol cm^-2^ s^-1^ with H_2_O_2_ and 469.56 pmol cm^-2^ s^-1^ with SNP treatments (**Figures 6A**–**8A**). Conversely, DMTU (a ROS scavenger, 5 mM), cPTIO (a NO scavenger, 300 μM), or PPADS (the antagonist of animal P2 receptors in the PM, 300 μM) significantly reduced NaCl-induced Na^+^ flux from *G. uralensis* roots (**Figures 6A**–**8A**). Our NMT data showed that the addition of agonists (H_2_O_2_, SNP, and eATP) or antagonists (DMTU, cPTIO, and PPADS) had no significant effect on Na^+^ flux in the absence of salt stress (**Figures 6A**–**8A**).

#### K^+^ Flux

Non-salinized *G. uralensis* roots displayed a stable, constant K^+^ efflux with a mean of 50.27 ± 8.49 pmol cm^-2^ s^-1^ (**Figure 4B**). Salt treatment markedly increased the K^+^ efflux, up to 151.35 pmol cm^-2^ s^-1^ in the measured regions (200–2700 μm from the apex) (**Figure 4B**). Inhibitor experiments showed that the salt-induced K^+^ loss was inhibited by a K^+^ channel blocker, TEA (**Figure 5C**). In contrast to TEA, sodium orthovanadate, the specific inhibitor of the PM H^+^-ATPase, markedly enhanced the salt-elicited K^+^ loss from liquorice roots (**Figure 5C**). This indicated that the K^+^ loss in salt-stressed roots was due to activation of DA-KORCs or NSCCs in the PM (Sun et al., 2009b; Zhang et al., 2015).

Of note, Ca^2+^, H_2_O_2_, SNP, or eATP reduced K^+^ efflux by 12–59% in salinized roots, although the effect H_2_O_2_ was more pronounced than that of the other agonists (**Figures 4B**, **6B**–**8B**). In contrast, salt-induced K^+^ efflux was significantly enhanced by all the tested antagonists, LaCl_3_, DMTU, cPTIO, and PPADS (**Figures 4B**, **6B**–**8B**). In general, none of the signaling molecules (Ca^2+^, H_2_O_2_, SNP, or eATP) or the inhibitors (LaCl_3_, DMTU, cPTIO, or PPADS) had a significant effect on K^+^ flux under no-salt control conditions (**Figures 4B**, **6B**–**8B**).

### Effect of Signaling Molecules on NaCl-Induced Transcription of K^+^/Na^+^ Homeostasis Genes

#### *GuAHA* and *GuSOS1*

**FIGURE 9:**
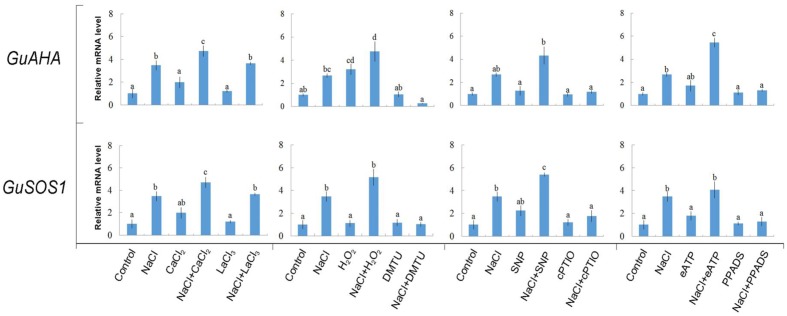
Effects of Ca^2+^, H_2_O_2_, SNP, eATP, and pharmacological agents on expression of *GuAHA* (PM H^+^-ATPase) and *GuSOS1* (salt overly sensitive 1 Na^+^/H^+^ antiporter) in *G. uralensis* roots under salt stress. Roots were exposed for 24 h to 0 or 100 mM NaCl, supplemented with or without CaCl_2_ (10 mM), H_2_O_2_ (10 mM), SNP (a NO donor, 100 μM), or ATP-Na_2_ (300 μM). Then, control and NaCl-stressed roots were treated with LaCl_3_ (5 mM), DMTU (5 mM), cPTIO (300 μM), or PPADS (300 μM) for 30 min. Quantitative RT-PCR results show the relative transcript abundance of *GuAHA* and *GuSOS1. GuActin2* served as the internal control for expression normalization. Forward and reverse primers for all tested genes are listed in Supplementary Table 1. Bars (±SD) represent the means of three to five individual plants; letters (a, b, c, and d) indicate significant differences between treatments (*P* < 0.05).

NaCl treatment (100 mM, 24 h) induced significant increases in the expression of Na^+^/H^+^ antiport system genes, *GuAHA* (PM H^+^-ATPase gene) and *GuSOS1* (PM Na^+^/H^+^ antiporter gene) (**Figure 9**). Interestingly, exogenously applied Ca^2+^, H_2_O_2_, SNP, or eATP increased the expression of *GuAHA* and/or *GuSOS1* under NaCl stress (**Figure 9**). These data suggested that Ca^2+^, H_2_O_2_, SNP, and eATP were involved in regulating the transcription of the PM Na^+^/H^+^ antiport system. Accordingly, pharmacological data showed that the salt-elicited upregulation of *GuAHA* and *GuSOS1* could be suppressed by DMTU, cPTIO, or PPADS (**Figure 9**). However, the Ca^2+^-channel inhibitor, LaCl_3_, did not block the salt-induced upregulation of *GuAHA* and *GuSOS1* transcription (**Figure 9**). Moreover, we found that these salt signaling molecules and pharmacological agents had no obvious effects on gene expression in the absence of NaCl stress, with the exception of H_2_O_2_, which induced *GuAHA* expression in control conditions (**Figure 9**).

#### Salt-Responsive Genes Related to Signaling Pathways

**FIGURE 10:**
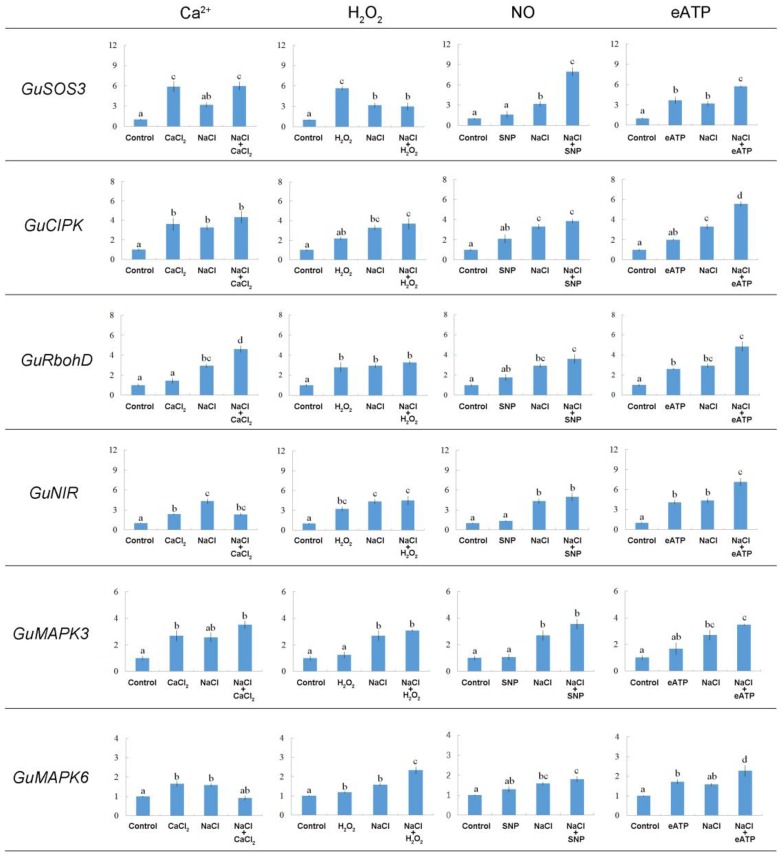
Effects of Ca^2+^, H_2_O_2_, SNP, and eATP on relative expression of salt-responsive genes in *G. uralensis* roots under salt stress. Roots were exposed for 24 h to 0 or 100 mM NaCl, supplemented with or without CaCl_2_ (10 mM), H_2_O_2_ (10 mM), SNP (a NO donor, 100 μM), or ATP-Na_2_ (300 μM). Quantitative RT-PCR results show the relative transcript abundance of homolog genes in *G. uralensis*, such as *GuSOS3* (salt overly sensitive 3), *GuCIPK* (CBL-interacting protein kinase), *GuRbohD* (respiratory burst oxidase homolog protein D), *GuNIR* (nitrate reductase), *GuMAPK3* (mitogen-activated protein kinase 3), and *GuMAPK6* (mitogen-activated protein kinase 6). Gu*Actin2* served as an internal control for expression normalization. Forward and reverse primers for all tested genes are listed in Supplementary Table 1. Bars (±SD) represent the means of three to five individual plants; letters (a, b, c, and d) indicate significant differences between treatments (*P* < 0.05).

As shown in **Figure 10**, NaCl increased the transcription of a series of salt-responsive genes. *GuSOS3* is important in Ca^2+^ signaling pathways (Zhu, 2001, 2003, 2016; Qiu et al., 2002; Yang et al., 2009; Ji et al., 2013); *GuCIPK* is important in Ca^2+^ signaling pathways (Xiang et al., 2007; Hu et al., 2015); *GuRbohD* is important in H_2_O_2_ signaling (Rejeb et al., 2015); *GuNIR* is important in NO signaling (Liu et al., 2007); and *GuMAPK3* and *GuMAPK6* are important in eATP signaling (Choi et al., 2014). We found that several signaling molecules changed the expression pattern of the selected salt-responsive genes under salt stress. For example, exposing NaCl-stressed plants to Ca^2+^, H_2_O_2_, or SNP enhanced transcription of *GuSOS3* or *GuCIPK* (**Figure 10**). Of note, eATP produced a pronounced induction of Ca^2+^ signaling pathway genes; the expression levels of both *GuSOS3* and *GuCIPK* were stimulated by eATP in NaCl-stressed roots (**Figure 10**). Also, *GuRbohD* transcription was enhanced by these signaling molecules, but Ca^2+^ and eATP produced more pronounced effects than H_2_O_2_ and SNP (**Figure 10**). *GuNIR* expression remained constant in NaCl-stressed roots, regardless of Ca^2+^, H_2_O_2_, or SNP treatment (**Figure 10**). However, *GuNIR* transcription was enhanced with eATP in salinized *G. uralensis* roots (**Figure 10**). The abundances of *GuMAPK3* and/or *GuMAPK6* transcripts increased in the presence of all signaling molecules, but H_2_O_2_ and eATP produced more pronounced effects on *GuMAPK6* (**Figure 10**). We also noticed that, in general, Ca^2+^, H_2_O_2_, and eATP increased the expression of the tested salt-responsive genes under no-salt control conditions; in contrast, SNP had less of an effect (**Figure 10**).

## Discussion

### NaCl Increased Salt-Sensitive Signaling Molecules in *G. uralensis* Roots

A short period of NaCl exposure caused cellular Na^+^ accumulation, which was more pronounced in roots than in leaves (**Figure 1**). The buildup of Na^+^ in root cells resulted in remarkable increases in cytosolic Ca^2+^, H_2_O_2_, NO, and eATP (**Figures 2**, **3**). The rapid increase of these signaling molecules indicated that *G. uralensis* roots could sense NaCl stress, and they set into motion a wide range of cellular processes required for salt adaptation (Chen and Polle, 2010; Chen et al., 2014; Polle and Chen, 2015). Accordingly, our NMT and RT-qPCR data revealed that eATP, Ca^2+^, H_2_O_2_, NO, and their interactions played crucial roles in regulating ion fluxes and gene transcription (**Figures 4**–**10**). These findings were similar to findings from our previous study in a salt-resistant poplar, *P. euphratica* (Sun et al., 2010a,b, 2012a).

### Signaling Molecules Upregulated Expression of the PM H^+^-ATPase and the Na^+^/H^+^ Antiporter

#### Na^+^ Homeostasis

To avoid toxicity, due to excessive Na^+^ in the cytosol, it is crucial for glycophyte plants to adapt to saline conditions (Shabala et al., 2005; Sun et al., 2009a,b, 2010a,b; Chen and Polle, 2010; Chen et al., 2014). The perennial species, *G. uralensis*, exhibited significant Na^+^ extrusion and a corresponding H^+^ uptake after exposure to 24-h NaCl treatments (**Figures 4**, **5**). However, the salt-induced Na^+^ efflux and H^+^ influx were markedly blocked by amiloride (an inhibitor of Na^+^/H^+^ antiporters) or sodium orthovanadate (a specific inhibitor of the PM H^+^-ATPase) (**Figure 5**). These results suggested that salinized roots of *G. uralensis* extruded Na^+^ and took up H^+^ via the activated Na^+^/H^+^ antiport system in the PM (i.e., the H^+^-ATPase and Na^+^/H^+^ antiporter; Shabala et al., 2003, 2005; Sun et al., 2009a; Lu et al., 2013; Lang et al., 2014; Zhao et al., 2016). Notably, we found that the Na^+^ efflux was enhanced by Ca^2+^, H_2_O_2_, NO, and eATP (**Figures 4**, **6**–**8**). Moreover, salt-induced Na^+^ extrusion could be reduced by pharmacological agents that blocked the pathways regulated by those molecules, i.e., LaCl_3_, DMTU, cPTIO, and PPADS, respectively (**Figures 4**, **6**–**8**). These results indicated that the signaling molecules were required to activate the PM Na^+^/H^+^ antiport system in the presence of NaCl salinity.

Our RT-qPCR assays showed that the activated Na^+^/H^+^ antiport system in salinized roots presumably resulted from the upregulation of *GuSOS1* and *GuAHA* genes (**Figure 9**). In a previous study, eATP was found to mediate the induction of *PeSOS1* and *PeAHA* in the poplar, *P. euphratica*, during NaCl stress (Sun et al., 2012a; Zhang et al., 2015). Moreover, NO was found to enhance Na^+^ exclusion by increasing the expression of the PM H^+^-ATPase and Na^+^/H^+^ antiporter in a secretor mangrove, *Avicennia marina*, under high salinity (Chen et al., 2010). Our previous study revealed that NO most likely interacted with Ca^2+^ and H_2_O_2_ in *Aegiceras corniculatum* to up-regulate the PM Na^+^/H^+^ antiport system (Lang et al., 2014). Chung et al. (2008) found that reactive oxygen species mediated SOS1 mRNA stability in Na^+^-treated Arabidopsis.

In addition to our agonist findings, the pharmacological data also showed that the salt-induced transcription of *GuSOS1* or *GuAHA* could be inhibited by DMTU, cPTIO, or PPADS in salt-stressed *G. uralensis* roots (**Figure 9**). These findings suggested that the endogenous salt-sensitive messengers, H_2_O_2_, NO, and eATP, contributed to the induction of *G. uralensis* Na^+^/H^+^ antiport genes during NaCl stress. However, the Ca^2+^-channel inhibitor, LaCl_3_, did not block the salt-responsive induction of *GuAHA* and *GuSOS1* (**Figure 9**). This result implied that vacuolar Ca^2+^ release might facilitate cytosolic Ca^2+^ signaling in the salt response of *G. uralensis* (Zhang et al., 2015). Indeed, in a previous study, we showed that a vacuole-generated Ca^2+^ signaling pathway participated in the regulation of ionic homeostasis in NaCl-stressed *P. euphratica* cells (Zhang et al., 2015).

#### K^+^ Homeostasis

In *G. uralensis* roots, NaCl-induced K^+^ efflux was blocked by TEA (a specific inhibitor of K^+^ permeable channels), but enhanced by vanadate (**Figure 5**). These findings suggested that NaCl-induced K^+^ loss was mediated by depolarization-activated channels, e.g., KORCs and NSCCs (Shabala et al., 2005, 2006; Sun et al., 2009b; Lu et al., 2013; Lang et al., 2014; Zhao et al., 2016). The addition of Ca^2+^, H_2_O_2_, NO, and eATP reduced the salt-induced K^+^ efflux (**Figures 4**, **6**–**8**). Presumably, this result was due to the inhibition of K^+^-channels by the activated PM H^+^-ATPase, because these signaling molecules upregulated *GuAHA* transcription in salinized roots (**Figure 9**). Previous studies have shown that NaCl-induced increases in PM H^+^-ATPase activity depended on H_2_O_2_ production, in *P. euphratica* (Zhang et al., 2007; Sun et al., 2010a,b) and in secretor and non-secretor mangroves (Lu et al., 2013; Lang et al., 2014). In *A. marina* leaves, NO remarkably enhanced PM H^+^-ATPase activity and *AHA1* transcription, and conversely, these activities were reduced by NO synthesis inhibitors and NO scavengers (Chen et al., 2010). The maintenance of K^+^ homeostasis in *P. euphratica* cells was attributed to the eATP induction of *AHA* (Sun et al., 2012a). Moreover, in poplar cells, NaCl-induced K^+^ loss increased, when *AHA* transcription was inhibited by the glucose-hexokinase trap system or P2 receptor antagonists (suramin and PPADS) (Sun et al., 2012a; Zhao et al., 2016). In the present study, we also found that NaCl-induced K^+^ loss increased (**Figures 4B**, **6B**–**8B**) and *GuAHA* expression was inhibited by the four tested antagonists, but LaCl_3_ produced less pronounced effects compared to DMTU, cPTIO, and PPADS (**Figure 9**). We concluded that salt-induced signaling molecules were required for upregulation of the PM H^+^-ATPase gene in *G. uralensis* roots. As a result, enhanced H^+^ pumping activity, on one hand, reduced K^+^ loss via depolarization-activated channels, and on the other hand, promoted Na^+^ extrusion via PM Na^+^/H^+^ antiporters (Chen and Polle, 2010; Chen et al., 2014; Polle and Chen, 2015).

### Multiple Signaling Networks Involved in the NaCl-Induced Expression of Salt-Responsive Genes Related to K^+^/Na^+^ Homeostasis

Clear interactions occurred between these stress signals to accelerate the transcription of salt-adaptive signaling pathway genes in *G. uralensis* roots. Ca^2+^ increased the *GuSOS3* expression (**Figure 10**), thus leading to enhanced Na^+^ extrusion via the SOS-signaling pathway (Zhu, 2001). In NaCl-treated roots of *G. uralensis*, H_2_O_2_, NO, or eATP, promoted the transcription of *GuSOS3*/*GuCIPK* (**Figure 10**), which indicated that these stress signals predominantly activated the Ca^2+^-SOS signaling pathway. A previous study in *P. euphratica* cells showed that exogenously applied H_2_O_2_ increased Ca^2+^ influx, which led to elevated cytosolic Ca^2+^ (Sun et al., 2010b). Based on our present results in *G. uralensis* roots, we suggest that H_2_O_2_ increased cytosolic Ca^2+^, which then mediated PM Na^+^/H^+^ antiport upregulation via the SOS-signaling pathway (Zhu, 2001, 2016). Furthermore, we found that NO enhanced the transcription of *GuSOS3*/*GuCIPK* in NaCl-stressed liquorice roots (**Figure 10**). Thus, NO-simulated Ca^2+^-SOS signaling would promote Na^+^ efflux and alleviate cellular Na^+^ toxicity in *G. uralensis*. Similarly, in the secretor mangrove, *A. corniculatum*, NO enhanced Na^+^ efflux elicited by Ca^2+^ (Lang et al., 2014).

Extracellular ATP signaling is a novel player in salt-stress acclimation. We found that eATP increased the expression of *GuSOS3, GuCIPK*, *GuRbohD*, *GuNIR*, *GuMAPK3*, and *GuMAPK6* (**Figure 10**). Moreover, *GuMAPK3* and *GuMAPK6* expression levels were enhanced by H_2_O_2_ in salinized *G. uralensis* roots (**Figure 10**). This indicated that eATP interacted with H_2_O_2_ and Ca^2+^ signaling to maintain K^+^/Na^+^ homeostasis. Previously, eATP was shown to interact with H_2_O_2_ and Ca^2+^ to increase Na^+^ extrusion in two mangrove species, *Kandelia obovata* and *A. corniculatum* (Lang et al., 2014). In *P. euphratica* cells, eATP signaling was mediated by H_2_O_2_ and cytosolic Ca^2+^ in the salt response (Sun et al., 2012a). Accordingly, eATP is thought to bind P2-like receptors in the PM (Choi et al., 2014), which leads to an increase in H_2_O_2_ and a transient elevation in cytosolic Ca^2+^ (Jeter et al., 2004; Demidchik et al., 2009; Sun et al., 2012a). Thus, eATP could initiate the H_2_O_2_ and Ca^2+^ signaling cascades and cause an increase in Na^+^/H^+^ exchange across the PM of *G. uralensis* roots under NaCl stress.

Extracellular ATP also increased the expression of *GuNIR* (**Figure 10**). This finding indicated that NO was a downstream component of eATP signaling. Similarly, in *P. euphratica* cells, NO was triggered by eATP, although NO played a negligible role in eATP-stimulated cell death (Sun et al., 2012b). There are species–specific interactions between eATP and NO in the mediation of K^+^/Na^+^ homeostasis (Lang et al., 2014). In this study, eATP signaling appeared to be mediated by NO in *G. uralensis* roots (**Figure 10**). However, in the non-secretor, *K. obovata*, NO was redundant in the presence of eATP, because eATP alone exerted a pronounced effect on Na^+^/H^+^ antiporters (Lang et al., 2014).

## Conclusion

**FIGURE 11:**
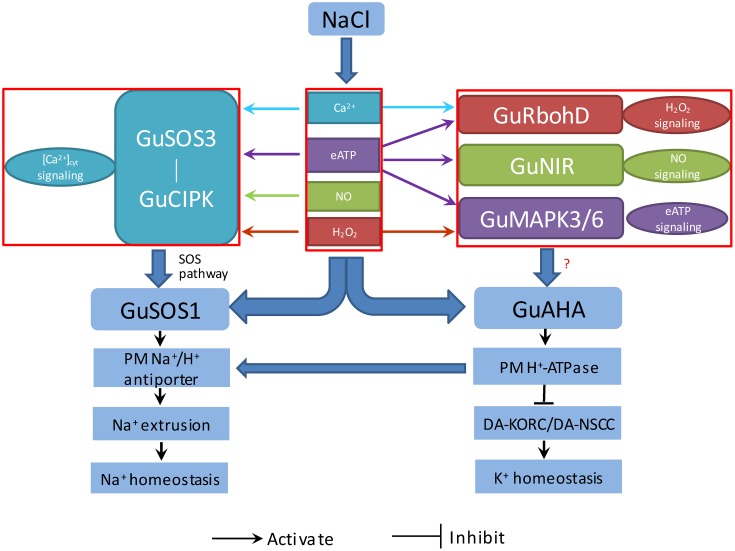
Proposed model of the signaling network (eATP, Ca^2+^, H_2_O_2_, and NO), which regulates salt-responsive genes, and their relationships to K^+^/Na^+^ homeostasis in *G. uralensis* roots. PM, plasma membrane; *G. uralensis* genes, *GuAHA*: PM H^+^-ATPase; *GuSOS1*, salt overly sensitive 1; *GuSOS3*, salt overly sensitive 3; *GuCIPK*, CBL-interacting protein kinase; *GuRbohD*, respiratory burst oxidase homolog protein D; *GuNIR*, nitrate reductase; *GuMAPK3*, mitogen-activated protein kinase 3; *GuMAPK6*, mitogen-activated protein kinase 6; Ion transporters: DA-KORCs, Depolarization-activated K^+^ outward rectifying channels; DA-NSCCs, Depolarization-activated non-selective cation channels.

Our findings suggested that salt exposure increased Ca^2+^, H_2_O_2_, NO, and eATP, which served as signaling molecules in mediating K^+^/Na^+^ balance by elevating Na^+^ efflux and restraining K^+^ loss in *G. uralensis*. Based on these results, we proposed a multiple signaling network for regulating ionic homeostasis in salinized *G. uralensis* (**Figure 11**). The NaCl-induced signaling molecules, Ca^2+^, H_2_O_2_, NO, and eATP, upregulated *GuSOS1* and *GuAHA* expression, which increased the numbers of Na^+^/H^+^ antiporters and H^+^ pumps in the PM. The enhanced Na^+^/H^+^ antiport system promoted the SOS-signaling pathway. In addition, H^+^-pump activity preserved the membrane potential, which restricted K^+^ efflux through DA-KORCs and DA-NSCCs. Interestingly, we also found interactions between these stress signaling molecules and the expression of salt-responsive genes in *G. uralensis* roots. Ca^2+^, H_2_O_2_, NO, and eATP enhanced *GuSOS3*/*GuCIPK* genes, which are related to the Ca^2+^-SOS signaling pathway. Moreover, eATP exhibited novel interactions with Ca^2+^, H_2_O_2_, and NO signaling, which contributed to the upregulation of *GuSOS3, GuCIPK*, *GuRbohD*, and *GuNIR*. This crosstalk was thought to contribute to the upregulation of *GuSOS1* and *GuAHA* expression in *G. uralensis* roots. Further investigations are needed to confirm these interactions.

## Author Contributions

TL, JX, and SC conceived of the original screening and research plans; SC supervised the experiments; TL, SD, NZ, CD, YnZ, YlZ, HZ, GS, and JY performed most of the experiments; CW, YW, QD, and SL provided technical assistance to TL, SD, and NZ; TL designed the experiments and analyzed the data; TL conceived of the project and wrote the article, with contributions from all the authors; SC supervised and complemented the writing. All authors have read and approved the manuscript.

## Conflict of Interest Statement

The authors declare that the research was conducted in the absence of any commercial or financial relationships that could be construed as a potential conflict of interest.

## Footnotes

1 http://www.youngerusa.com
