# Supplementary material for: Salt-Sensitive Signaling Networks in the Mediation of K+/Na+ Homeostasis Gene Expression in Glycyrrhiza uralensis Roots
Source: Front Plant Sci. 2017 Aug 14;8:1403. doi: 10.3389/fpls.2017.01403 (PMC5558103; doi:10.3389/fpls.2017.01403)
Supplement: Supplementary file 1 [file Image_1.pdf]

Supplemental TABLE 1 | Primer sets used for quantitative real-time PCR

Gene Name	GeneBank Accession NO. (*Glycyrrhiza uralensis*)	Arabidopsis Homolog locus	Primer sets	Sequence (5' to 3')
GuACT2	EU190972	AT5G09810	Forward primer	ATCACAATCGGAGCTGAGAG
			Reverse primer	ACTTTCTCTCTGGTGGAGCC
GuAHA	FS240345	AT5G57350	Forward primer	TATCGGTTATGGCAAGGTCA
			Reverse primer	GACCCCATCTGACTTCCCTC
GuSOS1	\	AT2G01980	Forward primer	AGCTAAGCACGATAATTGAAG
			Reverse primer	CATTCCTACAGCTCCAAGTG
GuCIPK	FS275069	AT5G35410	Forward primer	CGTCTACATGAGGTTCTGGC
			Reverse primer	GTCCGAAGCATACTAACCCC
GuSOS3	FS990589	AT5G24270	Forward primer	TCCTTGCTTCTGAAACACCC
			Reverse primer	AGATGCTCAAGGATCGGACA
GuRbohD	FS280550	AT5G47910	Forward primer	CTTGGCTAGCCCTTGGTTTA
			Reverse primer	ATTCCCAGGATAAACAGCCAC
GuNIR	FS239202	AT2G15620	Forward primer	TGTTCGATTTCTCACCCCTC
			Reverse primer	TAGCCAATTCATCAAGCCCT
GuMAPK3	FS269422	AT3G45640	Forward primer	GAGGTTACGGCCAAGTATCG
			Reverse primer	AAACTCTCTACGCAAGGGTG
GuMAPK6	FS279003	AT2G43790	Forward primer	ATACGAATGAGCATGTCGCC
			Reverse primer	ATTTGGTGAAGGTCGGTGTC
